# Assessment of Visual Reliance in Balance Control: An Inexpensive Extension of the Static Posturography

**DOI:** 10.1155/2014/248316

**Published:** 2014-02-19

**Authors:** Jozef Púčik, Marián Šaling, Tomáš Lukáč, Oldřich Ondráček, Martin Kucharík

**Affiliations:** ^1^Institute of Electronics and Photonics, Slovak University of Technology in Bratislava, Ilkovičova 3, 81219 Bratislava, Slovakia; ^2^II Neurologic Department, Medical Faculty of Commenius Univesity, Derer University Hospital, Limbová 5, 83305 Bratislava, Slovakia; ^3^Institute of Normal and Pathological Physiology, Slovak Academy of Sciences, Sienkiewiczova 1, 813 71 Bratislava, Slovakia

## Abstract

Ability of humans to maintain balance in an upright stance and during movement activities is one of the most natural skills affecting everyday life. This ability progressively deteriorates with increasing age, and balance impairment, often aggravated by age-related diseases, can result in falls that adversely impact the quality of life. Falls represent serious problems of health concern associated with aging. Many investigators, involved in different science disciplines such as medicine, engineering, psychology, and sport, have been attracted by a research of the human upright stance. In a clinical practice, stabilometry based on the force plate is the most widely available procedure used to evaluate the balance. In this paper, we have proposed a low-cost extension of the conventional stabilometry by the multimedia technology that allows identifying potentially disturbing effects of visual sensory information. Due to the proposed extension, a stabilometric assessment in terms of line integral of center of pressure (COP) during moving scene stimuli shows higher discrimination power between young healthy and elderly subjects with supposed stronger visual reliance.

## 1. Introduction

Functionality of the physiological systems deteriorates with aging. Declination in the functionality and performance of the systems, often aggravated by diseases which are commonly prevalent in elderly population, contributes to impairment of a balance. The balance-keeping ability, essential for daily life activities, requires the complex integration of sensory information about the position of the body relative to the surroundings and the ability to generate movement responses to correct the body position. The balance depends on contributions from vision, vestibular sense, proprioception, muscle strength, and cognition. Aging-related diseases, such as dementia, Parkinson disease, stroke, and Alzheimer disease, are linked with a balance deficit [1, 2]. The balance deficit is a predisposition to falls. Falls that occurred in elderly persons can be associated with a serious injury that adversely impacts the quality of life; even death is not uncommon as a final consequence of a fall.

Falls represent serious problems of health concern associated with aging. According to NIH/WHO report [3], based on compilation of studies carried out in Europe, “approximately 30% of people over 65 fall each year and for those over 75, the rates are higher. Between 20% and 30% of those who fall suffer injuries that reduce mobility and independence and increase the risk of premature death.”; “for women over 55 and men over 65, the age-specific death and admission rates for injury increase exponentially with age.” Therefore, prevention strategies for avoiding falls have to be properly studied. It is important to understand weak points in balance keeping mechanism and identify those people whose risk of falling is increased. Published studies have identified specific risk factors that include, among others, impaired mobility and gait, sensory deficit, and impaired cognition [3, 4].

Balance can be assessed by means of tests where an examiner assigns a score based on observation of examined person behavior during specific task, such as rise from chair, one leg stance, push-and-release test [5]. Other procedures require specific instruments to record biomechanical quantities, such as coordinates of the centre of mass (COM), torques, forces, and sway angles. Balance in quiet stance is commonly measured by force platform [6, 7]. The force platform is a rigid platform supported in 3 or 4 points in which load cells are located that produce signals used for calculation of COP coordinates. Notice that force platform measures coordinates of the centre of pressure (COP), that, in contrast to the centre of mass (COM) projected on the ground plane, involves contribution from body acceleration (or related forces).

Data provided by force plate can be utilized in two ways: “descriptive” and “model-based” approach. The former approach consists of calculation of stabilometric parameters, like mean velocities, rms values, and those based on nonlinear analyses. The later approach assumes a model of the postural system and identifies its parameters. From a biomechanical viewpoint, the human stance represents an unstable system, modeled as the inverted pendulum. In a simple model, the inverted pendulum is stabilized by hypothetical equivalent parameters: the spring stiffness and the damping [8]. A more realistic model is a feedback loop, with PID controller that generates the corrective torque according to sensory inputs to drive the body [9]. Advanced models include sensory dynamics and combination of individual sensory inputs (so-called sensory integration and related phenomenon sensory reweighting).

Effects of age on the postural control, postural parameters, and preferred strategies have been studied by researchers over the years [10]. Balance skills maturate, reaching optimum in adulthood, and decline in an old age. In terms of posturographic parameters that quantify imbalance (rms of COP values, mean COP velocities), curve of parameters as a function of the age is U-shaped, it reaches minimal values at adulthood, and marked increase starts after age of 60 [11]. Optimal values are reported inconsistently among studies, ranging from young adults [12] to age group 46–60 [13]. In perspective of balance control model, it was found that parameters of PID controller change: coefficients of proportional, derivative, and integral control increase with age [14]. In the study [14], these changes are interpreted as increased stiffness and damping that are closely related to proportional and derivative component of PID controller, respectively. Noise originated in sensory systems increases with age, leading to balance deterioration. Sensory reweighting due to aging has been also reported [15]. Though vision worsens with age, older people rely more on visual information. Role of the vision in postural control has several aspects. Not only reduced vision itself (visual acuity, contrast sensitivity, and depth cue perception), but also processing of visual information is important in balance control. Brain should correctly interpret optic flow to resolve motion ambiguity (self motion versus surrounding motion); it must cope with conflicting information from other sensory inputs and thus avoid improper postural response. Therefore, brain is loaded in balance control and concurrent cognitive task can interfere with balance control. Effect of this interference is stronger in older than young people, a phenomenon that can be investigated by so-called dual task paradigm [16].

Investigation of various aspects of postural control requires sophisticated experimental procedures. Conventional stabilometry needs to be extended by a stimulation procedure (moving scenes, galvanic stimulation, vibration, and controlled platform movement). This extension may require substantial changes in hardware and software solution. Complex measurement systems are available in specialized research laboratories, equipped with virtual reality environments. A commercially available instrument allowing exploration of some sensory integration aspects in postural control is known under name “EquiTest”; the related test is referred to as sensory organization test (SOT) [7].

In this work, we focus on visual sensory stimulation. Effects of visual motion stimuli on postural changes have been studied over past decades using various technologies. Majority of studies analyze posturographic parameters related to component corresponding to anteroposterior and mediolateral direction, or they quantify the stimulation effect in terms of parameters not specific to the sway direction. According to our knowledge, which type of motion stimuli is most effective to induce postural instability in visually dependent subjects, as well as optimal quantification of the effect, remains unsolved questions. In this paper, we propose configuration of experimental system that uses inexpensive commonly available devices. Existing stabilometric setting can be readily extended and used for investigation of a role of visual information in postural control and test visual dependence of a subject. In order to quantify effect of visual stimulation, we introduced velocity based parameters. We study effect of scene direction on anteroposterior (AP) and mediolateral (ML) components of mean velocities of COP. Effectiveness of parameters is evaluated in terms of discrimination ability between group of healthy young subjects and a group with increased visual reliance. The visually dependent group consists of seniors, including subjects with age related diseases.

## 2. Methods 

The measurement system is designed for recording postural responses to moving visual scene stimuli. Researchers use various technologies to elicit a visual stimulus, such as analog mechano-optical systems, computer-controlled mechanically driven moving patterns [17], rotating/tilting boxes or cylinders, or apparatuses based on digital technology, such as virtual reality environments [18, 19], with images projected on screens, HMD (head mounted displays), or special purpose projection systems, such as those known under recursive acronyms CAVE [20], NAVE [21] and BNAVE [22]. Advantage of mechanical systems is that they are not affected by phenomena and artifacts essential in digital technology, such as limited resolution, finite frame rate, aliasing, and dropped frames artifacts. On the other hand, a servomechanism is necessary and changes in scene type movement require construction changes. Special purpose projection systems (such as CAVE) are expensive and require extra space for installation and thus they are not suitable for standard clinical laboratories. In such situation, rear projection and HMD are options of choice. HMD displays suffer from problems, such as reduced field of view and require additional image preparation and adjustment for stereovision. In our system, we use commonly available multimedia technology—a projector and a rear projection screen, in a similar way as found in [23]. Unlike most researchers that use online generated scenes, we prefer precomputed stimuli encoded in a movie file and use single PC, which is responsible for stimuli presentation and measurement control.

Visual scenes were composed to induce body sway in 4 directions (AP scenes: forward, backward; ML scenes: left, right). Special attention was paid to avoid possibility of visual fixation; that is, no stationary point can be found in visible area of the moving scene. Constant velocity (linear or angular) stimulus was implemented.

### 2.1. Components of the System

**Figure 1:**
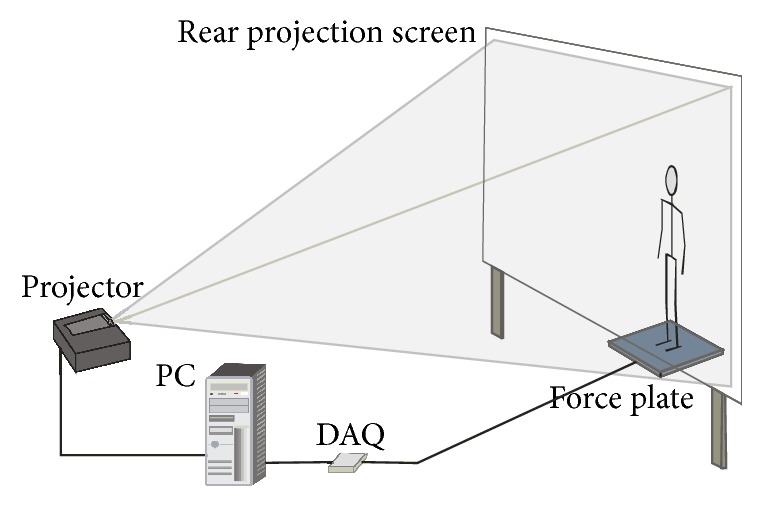
Outline of the experimental system.

The system includes the force platform, the data acquisition card, PC, the projector, and the back projection screen (Figure 1). The force platform used in our system was developed by Institute of Normal and Pathological Physiology, Slovak Academy of Sciences, and produces two analog signals proportional to deviations of COP in lateral (*x*-coordinate) and forward-backward (*y*-coordinate) directions. Signals are digitized at sampling rate 100 Hz by means of NI USB-6008, 12-bit data acquisition device. The whole measurement is controlled from Matlab environment, by means of the program that displays graphic user interface on primary monitor. The secondary monitor-projector is used for playing videostimulus, projected on the translucent screen. The projector is standard DLP type, with refresh rate of at least 60 Hz for smooth playing fast moving scenes at 60 fps, with native resolution 1024 × 768 points.

### 2.2. Visual Stimuli

Visual scenes used for moving stimuli were composed of 3D objects defined in VRML format (VRML97 standard). Moving scenes were created by translational and rotational movement of a camera (viewpoint) over a static scene, with constant linear and angular velocity, respectively. The stimuli movie files were produced in two ways: (1) controlling camera position in scene by Matlab script, implemented with help of Virtual Reality Toolbox (Matlab) and (2) using animation functionality inherent in VRML format. Camera position, look direction, and tilt are defined by three vectors in Virtual Reality Toolbox: *CameraPosition*, *CameraDirection*, and *CameraUpVector*. More detail on the scene composition and animation can be found in [24]. Animation defined in VRML uses *Interpolators* and *TimeSensor* nodes [25].

**Figure 2:**
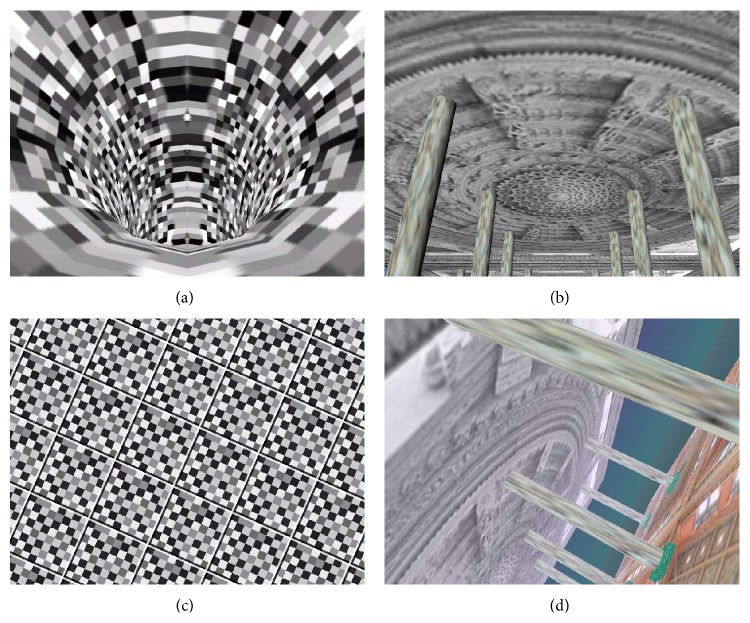
Designed visual stimuli: ((a), (b)) forward-backward direction; ((c), (d)) lateral direction.

Stimulation scenes are shown in Figure 2. Scenes (a) and (b) move in forward-backward direction at constant velocity; they are characteristic by expanding and contracting optic flow. Scenes (c) and (d) rotate clockwise or counterclockwise at constant angular velocity. The scenes (a), (c) have high contrast, while scenes (b), (d) have signs of visual polarity.

Rendered scenes were prepared in form of movie files and presented by Media Player Classic—Home Cinema (freeware) player. The player is controlled via web interface by commands delivered at specified times by measurement program.

### 2.3. Experimental Procedure

A participant in experiments underwent a measurement protocol, that consists of measurements of responses to 4 scenes of distinct directions (forward, backward, left, and right). Measured subjects were standing close to the projection screen (0.75 m); field of view was restricted by goggles to exclude visibility of projection screen frame. A single measurement starts with 10 s prestimulus period, and stimulation lasts for period of 10 or 20 s. Measurement is repeated 5 times in complete procedure to check consistency of responses and average results. Directions of the scenes were randomly shuffled to suppress subject adaptation to the scene. After two scenes were presented, the measurement was interrupted and subject relaxed for 2 minutes to avoid fatigue.

## 3. Results 

**Figure 3:**
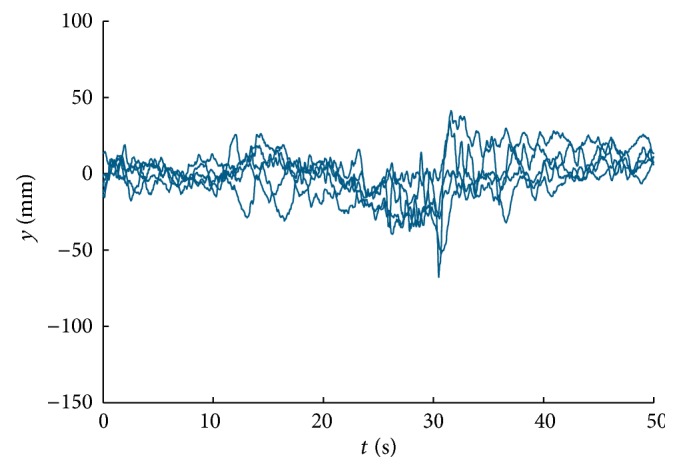
Responses to visual motion stimulus (AP stabilograms): young healthy subject, age 23, backward-moving scene.

**Figure 4:**
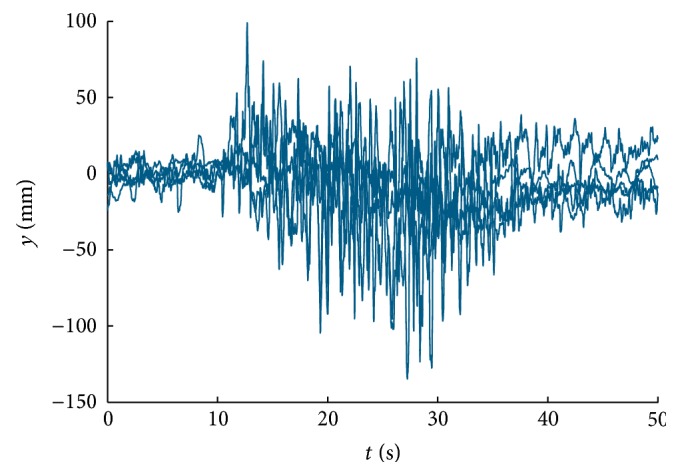
Responses to visual motion stimulus (AP stabilograms): subject with dementia, age 59, backward-moving scene.

In our previous work [24], we have proved that moving scenes depicted in Figures 2(b) and 2(d) induce postural sway and imbalance. We have found similar potential in scenes shown in Figures 2(a) and 2(c), especially in subjects with cognitive impairment. Representative responses measured in healthy young subject (age 23) and subject suffering from dementia (age 59) are shown in Figures 3 and 4, respectively. The responses recorded from subject with dementia manifest strong visual dependence of the subject; stimulation start time (10 s) and stop time (30 s) can be clearly identified in the figure. The subject sways in direction of stimulus motion.

Among experimental data acquired in volunteers, we have selected group of young healthy individuals (juniors, *N*_*j*_ = 5), age 20–30, and older age group, age 60–80 (seniors, *N*_*s*_ = 15). Balance of the subjects was quantified by parameter “line integral” (LI) that represents length of COP excursion during specific time interval (10 s in our measurements) [26]:

(1) $$\begin{array}{c} \text{LI}=\sum_{n=1}^{N-1}\sqrt{{\left(x\left[n\right]-x\left[n-1\right]\right)}^{2}+{\left(y\left[n\right]-y\left[n-1\right]\right)}^{2}}, \end{array}$$

where *x*[*n*], *y*[*n*] are digitized COP coordinates and *N* is number of data points in a data segment.

**Figure 5:**
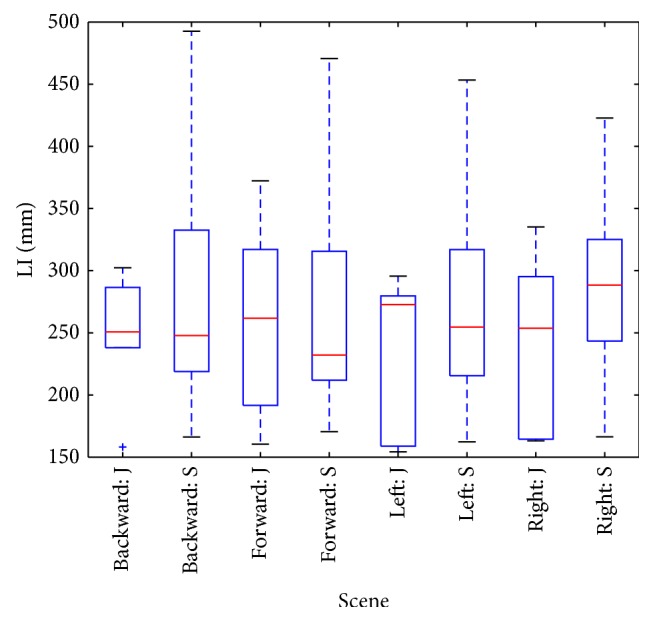
Box plot of prestimulus LI parameters: 4 scenes and two groups (juniors J, seniors S).

**Figure 6:**
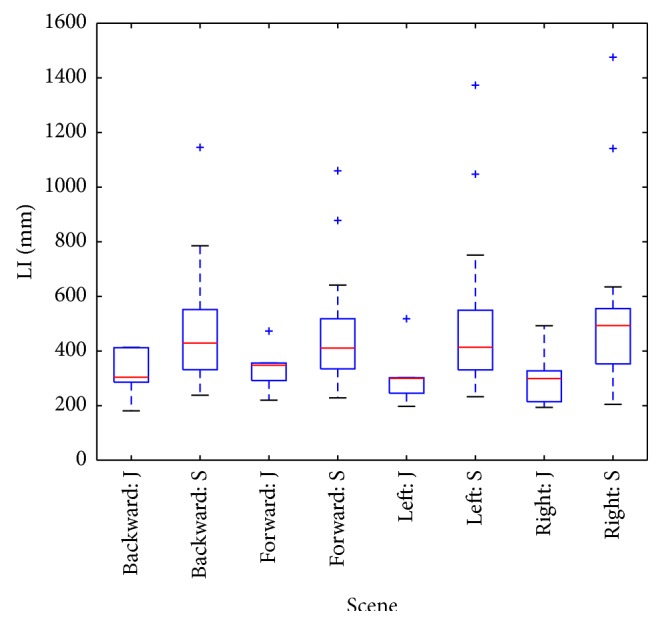
Box plot of intrastimulus LI parameters: 4 scenes and two groups (juniors J, seniors S).

**Figure 7:**
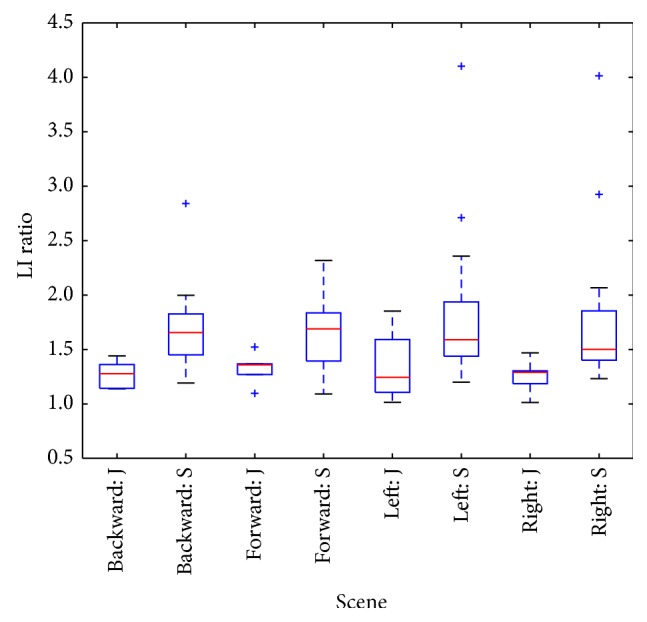
Box plot of LI intra-/prestimulus ratio: 4 scenes and two-groups (juniors J, seniors S).

Box-whisker plots of parameters (Figure 5, plotted by Matplotlib package in Python) indicate increased prestimulus LI values in senior group, but difference is not statistically significant (Table 1). Stronger distinction (Figures 6 and 7) between groups is expressed by LI parameter observed during stimulation and by LI ratio: intrastimulus LI (LI_1_) divided by its prestimulus value (LI_0_):

(2) $$\begin{array}{c} \text{L}{\text{I}}_{\text{ratio}}=\frac{\text{L}{\text{I}}_{1}}{\text{L}{\text{I}}_{0}}. \end{array}$$

Quantitative comparison of the groups is presented in Table 1, where between group difference is characterized by normalized mean difference of means: *t*-statistic assuming unequal variances

(3) $$\begin{array}{c} t=\frac{{\bar{m}}_{\text{LIS}}-{\bar{m}}_{\text{LI J}}}{\sqrt{s_{\text{LIS}}^{2}/N_{s}+s_{\text{LI J}}^{2}/N_{J}}}, \end{array}$$

where *m*_LIS_, *m*_LI J_ are arithmetic means, *s*_LIS_, *s*_LI J_ are sample standard deviations of particular LI parameter, and  *N*_*S*_,  *N*_*J*_  are sample sizes of senior and junior groups, respectively. *t*-test with Satterthwaite's approximation for the effective degrees of freedom was used in calculation of *P* values in Matlab package. In the case of visual stimulation, differences between groups reach (or approach to) statistical significance at 0.05 level.

Table 1 comprises also results for velocity parameters specific to either AP or ML directions. Mean velocity parameters *V*_*x*_, *V*_*y*_ in ML and AP directions are computed as

(4) $$\begin{array}{c} V_{x}=\frac{f_{s}}{N-1}\sum_{n=1}^{N-1}\left|x\left[n\right]-x\left[n-1\right]\right|,\\ V_{y}=\frac{f_{s}}{N-1}\sum_{n=1}^{N-1}\left|y\left[n\right]-y\left[n-1\right]\right|, \end{array}$$

where *N* is number of samples in analyzed segment, *f*_*s*_ is sampling frequency, and *x*[*n*] and *y*[*n*] are ML and AP components of stabilogram (COP coordinates). *V*_*x*_ and *V*_*y*_ are mean velocities in ML and AP directions, respectively. Effect of stimulation is expressed by normalized quantities separately in ML and AP directions

(5) $$\begin{array}{c} V_{x\;\text{ratio}}=\frac{V_{x1}}{V_{x0}},\\ V_{y\;\text{ratio}}=\frac{V_{y1}}{V_{y0}}, \end{array}$$

where indices 1 and 0 denote intrastimulus and prestimulus value of the velocity parameters, respectively.

Most clear distinction between groups is found in backward-moving scene, when LI_ratio_ is used as quantification parameter. Direction-specific parameters also show significant group differences, even if AP specific parameter is applied on ML scene and vice versa.

## 4. Discussion 

The purpose of this work was to provide an inexpensive procedure that can be implemented in a clinical laboratory in order to assess visual reliance of a subject. This kind of information can be useful in testing patient compliance to balance rehabilitation [27] or used in investigation of wide range of questions related to balance control, fall risk assessment, and prevention. We have used transient visual stimulus with constant velocity. Though postural responses to moving stimuli or optic flow are studied for years and robust responses are generally reported, we have encountered wide variability in sway waveforms. Sway direction of some subject was not concordant with moving stimuli direction or cannot be uniquely determined. After-effect, occurring when a stimulus is ceased [28], was not clearly observed in all subjects. Variability in waveforms complicates selection of such parameters that could reflect the waveform shape. On the other hand, line integral parameter, essentially equivalent to mean COP velocity, consistently shows increase due to stimulation in vast majority of the subjects for all moving scenes. In our previous study [24], effect of visual stimulation was more pronounced in LI parameter than RMS parameter. The LI parameter and additional velocity-based parameters were then used in quantitative description of postural responses in this work.

We have observed that LI postural parameter evaluated during stimulation in our experiments allowed better distinction between groups of juniors and seniors when comparison was based on measurements with the visual stimulation. We could not find statistically significant difference in LI parameter in prestimulus period, perhaps due to small sample size, but difference became significant for majority of scenes when visual stimulation was introduced. We have not applied any adjustments of postural parameters with respect to individual subject's heights or moments of inertia. In this regard, proposed LI ratio can be considered as a more reliable parameter, with reduced sensitivity to an anthropometric variability. Indeed, between group differences become more significant when LI ratio instead of intrastimulus LI parameters was used, (except left-rotating scene, Table 1).

**Figure 8:**
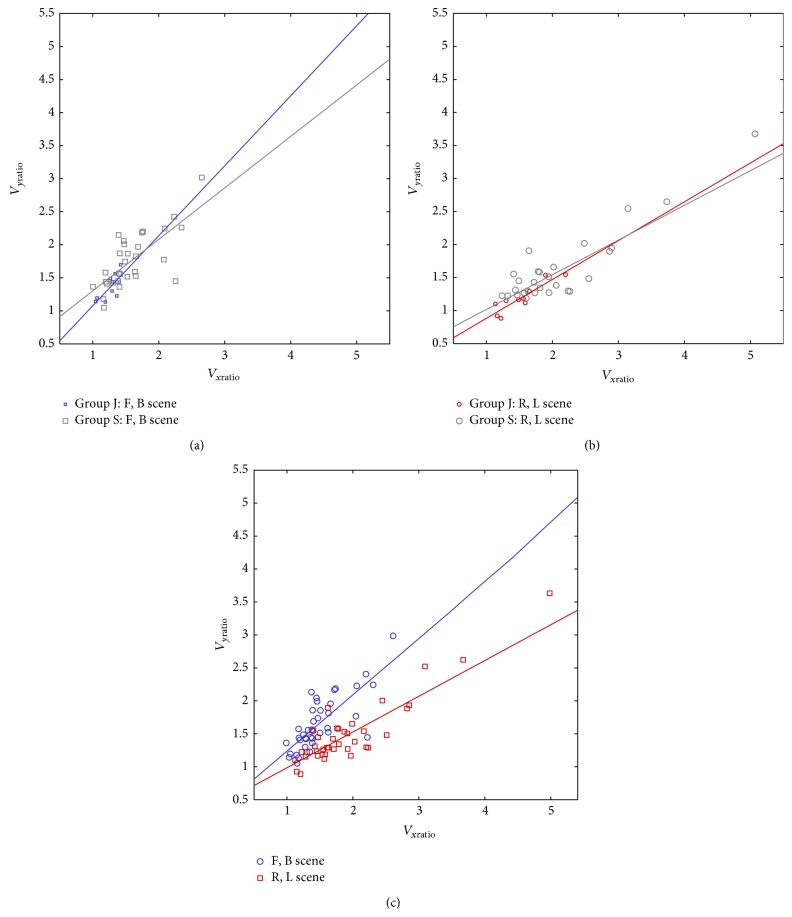
Analysis of slopes in AP versus ML parameters: (a) AP scenes and junior and senior group; (b) ML scenes and junior and senior groups; (c) AP and ML scenes, concatenated groups.

The line integral of COP comprises movements in both directions, AP and ML. In this work, we analyze the effect of a visual stimulus separately on ML and AP direction. An unexpected result is that a stimulus in one specific direction induces increase velocity parameters in the complementary direction with comparable amplitude; that is, AP moving scenes affect ML parameters and vice versa. A similar observation, expressed in terms of stabilogram standard deviations, was found in [23], but this study is restricted to roll motion scene and evaluated in vection period. These observations suggest dependence between AP and ML velocity parameters. We have performed correlation analysis (Pearson correlation coefficient, corrcoef in Matlab) of *V*_*x* ratio_ and *V*_*y* ratio_ that shows significant positive correlation in all scenes and both groups. Thereafter, we analyzed slopes of *V*_*y* ratio_ versus *V*_*x* ratio_ relations (Figure 8) by ANOCOVA (Matlab, aoctool). Between group differences are not significant by analysis (*F* = 0.17, *P* = 0.69 for AP scene; *F* = 0.07, *P* = 0.79 for ML scene), but we found significant difference in slopes between AP and ML scenes (*F* = 7.49, *P* = 0.008). The slope *V*_*y* ratio_/*V*_*x* ratio_ is higher in AP scene (0.85) than ML scene (0.54), as indicated in Figure 8(c). This finding is consistent with our expectation, that a direction-specific scene predominantly affects postural control in a respective direction.

Despite best performance (regarding group differentiation aspect) in our study exhibits backward-moving scene, we must be aware of individual sensitivity to the scene direction. After close examination of our data, we found that the highest *z*-scores (around 3.0) of LI parameter were identified in a subject with vascular dementia when ML stimuli were presented. But *z*-scores of this subject among AP stimuli are only below 0.5. Individual sensitivity of subjects to different visual stimuli was observed also in [29].

A potential limitation of our experimental data is that the senior group included apparently healthy individuals as well as those with mild form of age-related diseases, such as incipient dementia, that can contribute to presence of outliers seen in presented graphical results. Total number of subjects with dementia (vascular, Alzheimer, mixed or Prick's types) was 8. Differentiation between healthy seniors, affected by natural aging, and those affected by age-related diseases is another question of scientific interest [11].

## 5. Conclusion 

Force platform stabilometry is the measurements procedure that quantifies balance impairment, for example, in elderly and diseased subjects. In this work, we have presented a complementary procedure that has potential to be more sensitive in detection of balance changes due to aging and age-related diseases. We propose configuration of experimental system that uses inexpensive commonly available multimedia technology, data acquisition devices, and single PC. Since commercial force plates usually provide also analogue outputs, extension of existing posturography systems is possible.

It is known that the vision plays an important role in the balance control. Our system uses visual motion stimulus as a destabilization factor. In order to elicit the stimuli, we have implemented an extension of the clinical stabilometric laboratory as an alternative solution to appliances available in research laboratories. The proposed system is suitable to study differences in visual information role between groups of healthy individuals and those impaired by aging and/or specific diseases. Analysis of measurements demonstrated enhancement of between-group difference due to visual stimulation that proves merit of the proposed extension in posturographic assessment. After development of a proper diagnostic procedure, it could be possible to identify visually-dependent individuals, which may be at increased risk of a fall due to instability in presence of a misleading moving visual cue occasionally encountered in everyday life.

## Figures and Tables

**Table 1:** Between group difference in postural parameters.

*t*-statistic (*P* value)	Scene type			
	Backward	Forward	Left	Right

LI_0_	0.92 (*P* = 0.37)	0.32 (*P* = 0.76)	1.00 (*P* = 0.34)	1.07 (*P* = 0.32)
LI_1_	2.18 (*P* = 0.043)	1.78 (*P* = 0.092)	2.07 (*P* = 0.053)	2.31 (*P* = 0.033)
LI_1_/LI_0_	3.54 (*P* = 0.002)	2.88 (*P* = 0.011)	1.94 (*P* = 0.071)	2.72 (*P* = 0.014)
*V*_*x*1_/*V*_*x*0_	3.04 (*P* = 0.007)	2.43 (*P* = 0.026)	1.86 (*P* = 0.082)	2.61 (*P* = 0.019)
*V*_*y*1_/*V*_*y*0_	3.32 (*P* = 0.004)	2.41 (*P* = 0.031)	2.15 (*P* = 0.047)	3.03 (*P* = 0.007)

LI_0_—prestimulus LI parameter.

LI_1_—intrastimulus LI parameter.

*V*_*x*0_, *V*_*y*0_—prestimulus mean velocity in ML and AP direction.

*V*_*x*1_, *V*_*y*1_—intrastimulus LI parameter in ML and AP direction.
